# Nuclear magnetic resonance spectroscopy and infrared spectroscopy analysis of precipitate formed after mixing sodium hypochlorite and QMix 2in1

**DOI:** 10.1371/journal.pone.0202081

**Published:** 2018-08-15

**Authors:** Özgür Irmak, Ekim Onur Orhan, Kamuran Görgün, Batu Can Yaman

**Affiliations:** 1 Department of Restorative Dentistry, Faculty of Dentistry, Eskişehir Osmangazi University, Eskişehir, Turkey; 2 Department of Endodontics, Faculty of Dentistry, Eskişehir Osmangazi University, Eskişehir, Turkey; 3 Department of Science, Faculty of Chemistry, Eskişehir Osmangazi University, Eskişehir, Turkey; Northeastern University, UNITED STATES

## Abstract

**Background:**

Mixing sodium hypochlorite (NaOCl) with chlorhexidine (CHX) forms a brown precipitate. QMix-2in1 (QMix) was introduced as a final irrigant. Manufacturer recommends interim flushing with saline between the application of NaOCl and QMix to prevent formation of precipitation. This study assessed whether para-chloroaniline (PCA) is formed after mixing NaOCl with QMix.

**Methods:**

Commercially available, 5.25% NaOCl solution, 2% CHX, QMix, 15% ethylenediaminetetraacetic acid (EDTA) and 98% PCA in powder form were used. Groups were prepared at room temperature. Group 1, 98% PCA in powder form; Group 2, 2% chlorhexidine (CHX); Group 3, QMix; Group 4, 5.25% sodium hypochlorite (NaOCl) mixed with QMix; Group 5, 5.25% NaOCl mixed with CHX; Group 6, 15% EDTA mixed with CHX. The precipitates were extracted and analysed with Proton Nuclear Magnetic Resonance (1H-NMR) and Infrared (IR) Spectroscopy, using PCA as an internal standard.

**Results:**

No PCA was found in any of the irrigant-mixture groups tested.

**Conclusions:**

This study used the interpretation of spectral results for the amino signals of precipitate formed after mixing QMix with 5.25% NaOCl using different nondestructive analysis methods, with PCA as an internal standard (control). We conclude that mixing QMix or 2%CHX with 5.25% NaOCl does not yield free PCA.

## Introduction

Microorganisms can lead to development of pulp and periradicular infections [1]. The main goal of root canal treatment is to eliminate the microorganisms and their products from the root canal system [1]. Root canal irrigation, along with mechanical cleaning plays an important role for success in endodontic therapies [2]. Sodium hypochlorite (NaOCl) is one of the most frequently used root-canal irrigation solutions during endodontic therapy. It has antimicrobial properties and organic tissue dissolving capacities [3]. However, in high concentrations NaOCl is toxic and may irritate the periradicular tissues [4]. Chlorhexidine digluconate (CHX), a broad-spectrum antimicrobial agent, is also used for root canal irrigation for root canal disinfection[5], but has no tissue dissolution capacity [6]. Different irrigation solutions can be used in combination with NaOCl to enhance the antimicrobial effect of the irrigation procedure [7, 8]. However, caution should be taken when using NaOCl and CHX together. Before introducing CHX into the root canal, NaOCl should be completely removed from the canal system. Somehow if these two irrigation solutions are mixed a brown-colored precipitate is formed. Removal of this precipitate from the canal system may be difficult and discoloration of the dental structure may occur [9]. The structure of this brown precipitate has not been fully elucidated. Previously, it was suggested that the brown precipitate formed after mixing NaOCl and CHX, contains para-chloroaniline (PCA) [10–13]. PCA, is an aniline derivative, which is known to be toxic [14] and carcinogenic [15]. However, two studies utilizing proton nuclear magnetic resonance (1H-NMR) analysis [16, 17], and another study utilizing 1H-NMR and multiple other spectroscopy techniques [18], showed that there is no free PCA in this brown precipitate.

QMix 2in1 (Dentsply Tulsa Dental, Tulsa, OK, USA) (QMix) was introduced in 2011 as an endodontic irrigation solution. QMix is an aqueous solution containing EDTA, CHX analog and N-cetyl-N,N,N-trimethylammonium bromide (CTAB) [19]. It serves as a broad-spectrum antimicrobial and smear layer removal agent in a single application and is used as a final irrigant after NaOCl irrigation. Manufacturer of QMix recommends an interim flushing with saline between the application of NaOCl and QMix to prevent formation of precipitation and brown discoloration as a result of these two solutions’ interaction [20]. When saline is used between NaOCl and QMix application, no precipitate is formed[11]. In the literature there are very few studies which investigated the interaction of NaOCl with QMix.

A recently published study, utilizing 1H-NMR suggested that the brown precipitate formed after mixing NaOCl with QMix did not contain PCA, while mixing 2.5% NaOCl with 2% CHX yielded PCA [21]. Since the authors did not use standard PCA as control in their study, their results may not be fully reliable. Therefore, this study aimed to determine whether PCA is formed after mixing 5.25% NaOCl with QMix by using 1H-NMR spectroscopy.

## Materials and methods

Commercially available, 5.25% fresh NaOCl solution (Chlorax 5.25, Cerkamed Medical Company, Stalowa Wola, Poland), 2% CHX (Gluco-Chex solution, Cerkamed Medical Company), QMix (Qmix 2in1, Tulsa Dental, Dentsply, OK, USA), 15% ethylenediaminetetraacetic acid (EDTA) (Endo-solution, Cerkamed Medical Company) and 98% PCA (4-Chloroaniline, Sigma-Aldrich, St. Louis, MO, USA) in powder form were used.

### 1 Preparation of solutions

Total of twenty ml of solution was used for each group. Ten ml per irrigant (total of 20 ml) was used in groups with two mixed irrigants (Table 1). Solutions were mixed in glass beakers. Delivery of additional solution and mixing (Stirring Rod, Acrol Scientific Laboratory Systems, İstanbul, Turkey) were completed in 5 s at room temperature (25°C).

**Table 1:** Experimental groups.

**Groups**	**Solution**	**Additional solution**	**Precipitate color**
**1**	PCA	-	No precipitate
**2**	QMix	-	No precipitate
**3**	CHX	-	No precipitate
**4**	NaOCl	QMix	Brown precipitate
**5**	NaOCl	CHX	Brown precipitate
**6**	EDTA	CHX	Milky white precipitate

Abbreviations: PCA, para-chloroaniline; CHX, chlorhexidine; NaOCl, sodium hypochlorite; EDTA, Ethylenediaminetetraacetic acid

### 2 Extraction and/or solvation

Analytical grade ethyl acetate was used for extraction and/or solvation. The solution yielding a precipitate was extracted three times in 15 mL of ethyl acetate to transfer the precipitates into organic solvent. Organic layer was separated and dried over anhydrous sodium sulphate. Solvent was removed under reduced pressure and solid brown precipitates with different weights (masses) were obtained [18]. These solids were used for 1H-NMR spectroscopy analyses. QMix-only, and CHX-only solutions were lyophilized for elimination of broad water signal from 1H-NMR spectra.

### 3 Proton Nuclear magnetic resonance (1H-NMR) Spectroscopy

1H NMR experiments were performed in Bruker AVANCE 500 Spectrometer (Bruker UltraShield 500 plus, Bruker, Billerica, MA, USA) using 5 mm BBO probe at 298 K. Field strength was 11.7 Tesla. The operating frequencies were 500.13 MHz and for 1H NMR. 1H-NMR spectra of lyophilized CHX, QMix, brown precipitates and PCA were recorded in perdeuterated dimethyl sulfoxide (d6-DMSO) separately in accordance with tetramethylsilane as an external standard in a 1H-NMR Spectroscopy Unit [18].

### 4 Infrared (IR) Spectroscopy analysis

IR spectra were recorded using potassium bromide (KBr) disc technique in the region 4000–400 cm^-1^ (PerkinElmer IR Spectroscopy, Waltham, MA, USA). The spectral resolution was ±1 cm^-1^.

Both 1H-NMR and IR analyses were run in triplicates for each experimental group.

## Results

**Fig 1:**
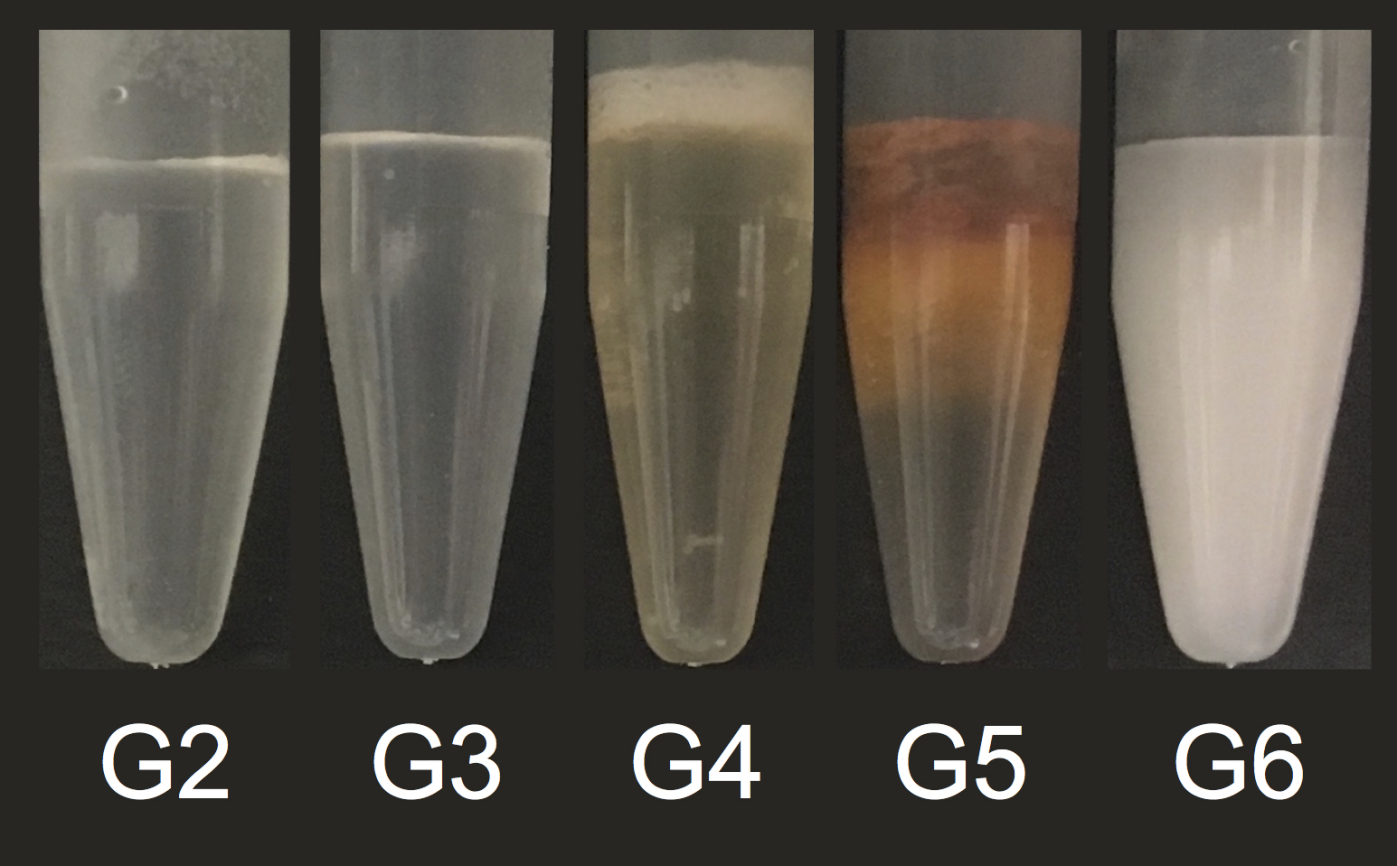
Appearances of the solutions prepared: G2, 2% chlorhexidine (CHX); G3, QMix; G4, 5.25% sodium hypochlorite (NaOCl) mixed with QMix; G5, 5.25% NaOCl mixed with CHX; G6, 15% Ethylenediaminetetraacetic acid (EDTA) mixed with 2% CHX.

**Fig 2:**
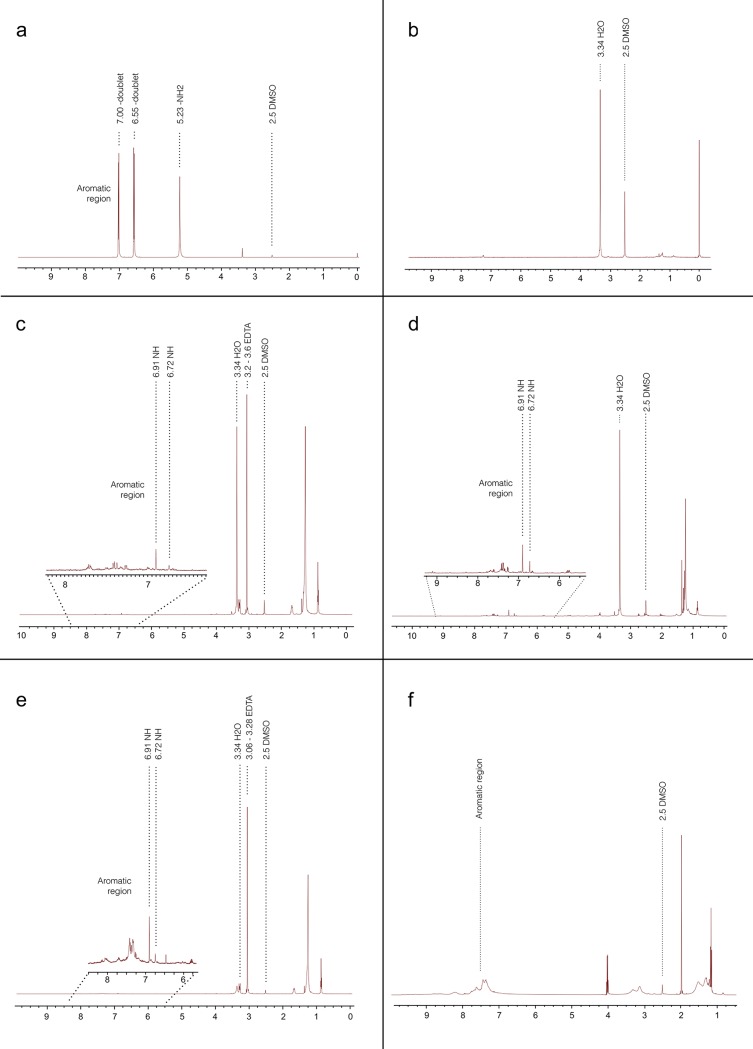
1H NMR spectra of groups; a: PCA, b: EDTA + CHX, c: QMix, d: CHX, e: NaOCl + QMix, f: NaOCl + CHX. **Abbreviations:** PCA, para-chloroaniline; CHX, chlorhexidine; NaOCl, sodium hypochlorite; EDTA, Ethylenediaminetetraacetic acid.

**Fig 3:**
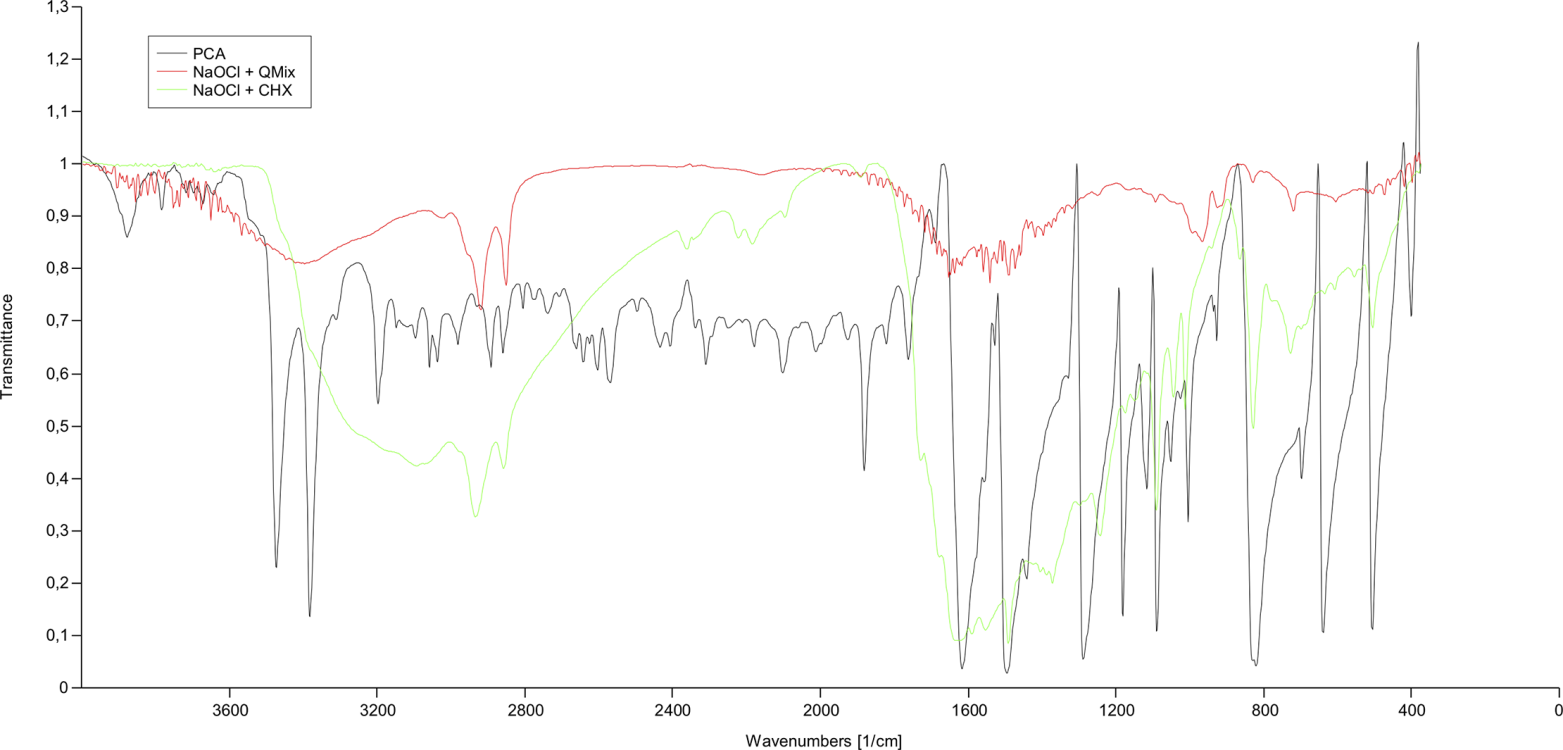
IR spectra of brown precipitates and PCA. **Abbreviations:** PCA, para-chloroaniline; CHX, chlorhexidine; NaOCl, sodium hypochlorite.

The color appearances of the solutions were as follows: Group 2 (QMix) and Group 3 (CHX) were colorless; Group 4 (NaOCl+QMix) and Group 5 (NaOCl+CHX) were brownish; Group 6 (EDTA+NaOCl) was milky-white (Fig 1 and Table 1). 1H-NMR spectra of PCA, CHX, QMix and precipitates are shown in Fig 2. IR spectra of all precipitates and PCA are shown in Fig 3.

In 1H-NMR spectrum, standard PCA yielded two doublet signals at 7.00 and 6.55 ppm with coupling constant of J = 8.65 Hz for aromatic protons on benzene ring and one singlet signal for amino (-NH_2_) group. In spectra of other groups, chemical shifts were recorded between 7.30–7.80 ppm, which were different than aromatic signals of standard PCA.

For standard PCA, IR spectroscopy showed asymmetric -NH_2_ stretch at 3472 cm^-1^ and symmetric stretch at 3381 cm^-1^, respectively. Additionally, a scissoring signal of -NH_2_ group at 1617 cm^-1^ was recorded. None of these -NH_2_ signals were recorded for any other group. IR spectra of Group 4 (NaOCl+QMix) yielded–OH signals at 3246 cm^-1^ instead of–NH_2_ signals, confirming the absence of free PCA.

1H-NMR and IR spectroscopy analyses showed that no free PCA was present in CHX, QMix and precipitates.

## Discussion

NMR spectroscopy can be used to analyze molecules in mixtures without fragmentation of the molecules. In this method, peaks of the spectrum pattern (singlet, doublet, etc.) of the mixture and standard sample are compared. If same spectrum patterns are at the same chemical shift, then the investigated sample is confirmed to be present in mixture. In 1H-NMR spectra aromatic groups yield signals between 7 and 10 ppm. Free PCA has characteristic primary amino (–NH_2_) moiety at 5.5 ppm. Presence of amino (–NH_2_) signal indicates presence of free PCA. As previously showed [18], and recently confirmed in this study, 1H-NMR spectra of mixture groups had different chemical shift values (7.30–7.80 ppm) than aromatic signals of standard PCA (7.00 and 6.55 ppm). These differences were also observed in the IR spectrums confirming the absence of free PCA in none of the brown precipitates.

CHX forms a salt mixed with EDTA resulting from acid-base reactions and a milky white precipitate is produced [22]. We also analyzed mixture of EDTA and CHX as a separate group (Group 6), since they are also present in QMix. In Group 6 (EDTA+CHX), no brown precipitate was formed; instead cloudy light grey colored precipitate was observed. No solid part of this mixture could be transferred into organic phase by extraction process with ethyl acetate. This indicated that mixture remained in water phase. After removal of water, obtained solid mass was analyzed with IR spectroscopy. IR spectra showed no characteristic peaks. This solid was then transferred into d6-DMSO solution for 1H-NMR analysis. Spectra yielded peaks for only d6-DMSO and water for Group 6 (EDTA+CHX), indicating that mixture couldn’t be dissolved in d6-DMSO. In Group 2 (QMix), contrary to Group 6 (EDTA+CHX), it was possible to transfer the solution to organic phase since QMix could be dissolved in d6-DMSO. In the NMR spectra, there were no peaks of PCA, but we observed peaks related to EDTA (3.06–3.28 ppm), water (3.34 ppm), d6-DMSO (2.5 ppm). We speculate that QMix’s transfer to organic phase was possible with the help of CTAB. According to the patent [19], when preparing QMix, mixing CTAB with CHX in water prior to addition of EDTA makes it possible to prevent precipitation that occurs as a result of interaction between CHX and EDTA. For this reason, it could have been possible to analyze Group 2 (QMix) but not Group 6 (EDTA+CHX) in NMR.

Several previous studies investigated the presence of PCA in NaOCl-CHX mixture [10–13], and NaOCl-QMix mixture [11, 21]. Studies that employed mass spectroscopy (MS) to analyze the precipitate formed after mixing CHX and NaOCl found PCA in the mixture [10–13]. Since MS could lead to fragmentation of the investigated molecules one could get misleading results [18]. It could be more appropriate to use non-destructive methods such as 1H-NMR. We found no free PCA in any of NaOCl-CHX and NaOCl-QMix mixtures in this study. Our results are in agreement with the previous studies which used 1H-NMR and did not find free PCA in the brown precipitate formed after mixing NaOCl and CHX [16–18]. Until recently, two studies investigated the reaction between QMix and NaOCl [11, 21]. In one study, the reactions of NaOCl+CHX and NaOCl+QMix were investigated. Authors found lower amount of brown precipitate after mixing NaOCl with QMix when compared with the brown precipitate formed after mixing NaOCl with CHX. They attributed this result to lower amounts of CHX present in QMix. Our results, regarding the amount of brown precipitate (Fig 1) are in agreement with the mentioned study. Additionally, they found that mixing NaOCl with CHX produced PCA, whereas mixing NaOCl with QMix did not. Authors have interpreted the spectrum between 6.5–8.5 ppm and stated that recorded peaks at 7.0–8.0 ppm represented PCA in the mixture. However, 1H-NMR analysis of standard PCA for comparison was not included. To be able to state that PCA is formed from reaction of CHX and NaOCl by using 1H-NMR analysis, it is vital to record an amino (-NH_2_) signal at 5–5.5 ppm. Nevertheless, abovementioned study neither discusses nor gives any spectral result for the amino signal. Therefore, their finding about occurrence of free PCA in the mixture may be inaccurate. Moreover, in practice it may not be easy to transfer very low amounts of precipitate to organic phase and subsequently analyze it in 1H-NMR spectroscopy. As a consequence, we speculate that their results are proof for only para-chloro amido moiety of CHX or any derivative of CHX in brown precipitate, but not for free PCA. In the other study, no precipitate or PCA was detected on dentin irrigated with NaOCl and QMiX [11]. However, that particular study saline was used as an intermediate flushing agent between NaOCl and QMix; therefore, its methodology and results do not correspond to that of our study.

Previous studies analyzed CHX in chromatography-mass spectrometry (GC/MS), and a signal at 127 m/z that corresponds to PCA, was registered [18, 23]. GC/MS leads to separation of the analyzed samples by ionization [24]. PCA is possible fragment of CHX in ionization process during GC/MS and could mislead the results regarding the formation of PCA [18]. In this study non-destructive methods such as 1H-NMR and IR spectroscopies were used and PCA was not found in any of the mixtures analyzed. However, the exact nature of the precipitate could not be elucidated by this study. It is possible that isomers of PCA could have been produced which are known to be hemotoxic [14, 25] and carcinogenic [15]. Until the composition the precipitate is fully investigated, clinicians should avoid mixing these solutions since it was shown to be present on root canal walls [9, 11], and difficult to remove [26] and could have a negative impact on the outcome of the root canal treatment.

## Conclusions

This study interpreted the spectral results for the amino signals of precipitate formed after mixing QMix with 5.25% NaOCl using PCA as an internal standard for the first time. In conclusion, this study did not find free PCA in the precipitate formed after mixing QMix or 2% CHX with 5.25% NaOCl. Further studies are needed to assess the molecular structure of the brown precipitate.
